# Examining the mechanics of rope bending over a three-dimensional edge in ascending robots

**DOI:** 10.1038/s41598-023-48078-5

**Published:** 2023-11-24

**Authors:** Myeongjin Choi, Sahoon Ahn, Hwa Soo Kim, Taewon Seo

**Affiliations:** 1 School of Mechanical Engineering, Hanyang University, Seoul, 04763 Republic of Korea; 2 Department of Mechanical System Engineering, Kyonggi University, Suwon, 16227 Republic of Korea

**Keywords:** Engineering, Mechanical engineering

## Abstract

This paper presents the analysis of ropes’ bending on three-dimension edges by ascending robots. A rope ascending robot (RAR) is a type of exterior wall-working robot that utilizes a synthetic rope to traverse the outer surface of a building. Rope-based façade cleaning robots demonstrate effective performance in well-structured buildings. However, in unstructured buildings, the rope used by these robots may become entangled or caught on various structures, presenting a significant challenge for their operation. If the rope becomes caught on a structure, the robot will be unable to move to its intended position. In more severe cases, the rope may become damaged, leading to potential failure or even a fall of the robot. Therefore, solving this problem is crucial for safe and efficient robot operation. Consequently, this study defines the issue of the rope becoming caught on a structure as a rope-locking problem and analyzes it by categorizing it based on the dimensions of contact between the rope and the edge. To address the varying tension experienced in different areas, the rope was divided into micro units and subjected to a three-dimensional analysis to resolve the rope-locking problem. Additionally, the analysis was verified by experiments.

## Introduction

Modern buildings are becoming increasingly diverse with the development of architectural technologies. Modern buildings are designed to be more aesthetically pleasing than existing buildings. The use of ropes for building maintenance and repair can pose a significant risk of accidents for workers. This is due to the fact that the method used for maintenance and repair typically involves workers climbing the building directly using ropes.

**Figure 1:**
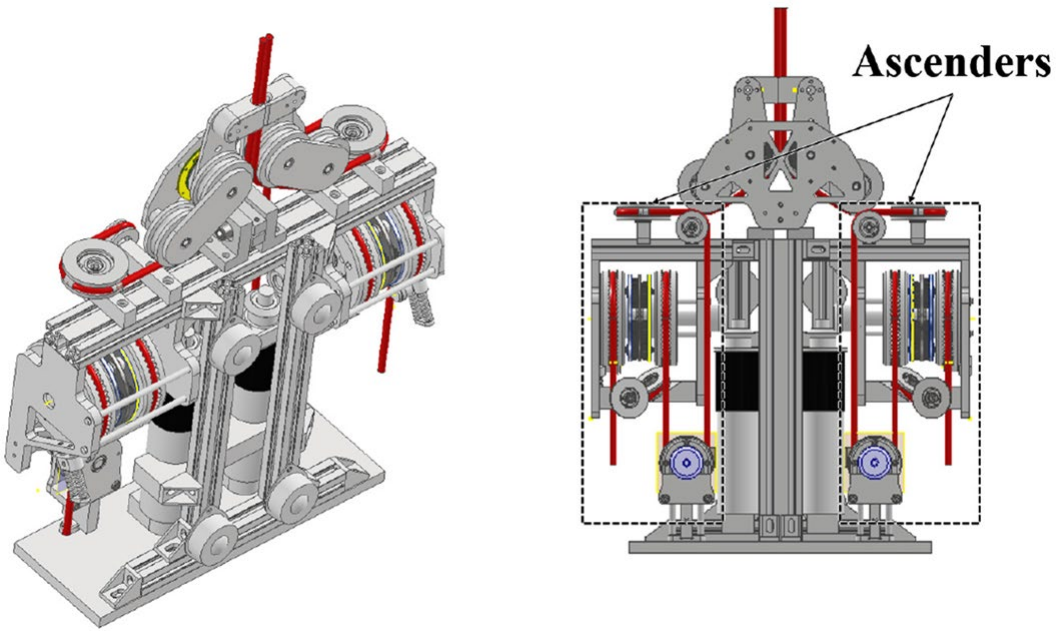
Overall appearance of the rope ascending robot.

This study aimed to address the safety concerns associated with building maintenance and repair by developing a rope-ascending robot (RAR) (Fig. 1). The RAR is equipped with robot-embedded ascenders, which allows it to move freely on structured buildings using two fiber ropes. This is different from other robots used for building maintenance and repair, such as dual-rope winch robots^1^, two-rope-driven mobile robots^2^, and dual-ascender robots^3,4^, which rely on an external winch. However, the use of ropes in the RAR presents a limitation for its application in unstructured buildings, where the lack of anchor points and support structures for the ropes makes it challenging to use the robot.

Specifically, support structure can makes RAR critical problems. RAR hangs from a building by tying end of the rope to a fixed anchor and winding other end to robot respectively. RAR’s operation characteristic can cause the rope to become lock onto its support structure that existed in rope path. This phenomenon is usually caused by the frictional force generated when the rope comes in contact with the support structure due to rope’s bending. Rope may cut by accumulated damage in the rope and rope’s cut can cause robot’s falling under the building in severe cases. However, using a separate control technique for RAR could potentially enable its application in unstructured buildings.

A separate control technique for RAR’s application in unstructured buildings was named Rope Impact Control (RIC). When rope is locked on support structure of unstructured buildings, the rope reaches force equilibrium and also forms a specific angle according to the rope path. So, if rope’s contact angle with structure can be change to reach force disequilibrium, rope can be unlock from lock state. In short, RIC is a control that take advantage of force disequilibrium uses impact to change rope’s contact angle with support structure. However, in order to implement RIC to RAR, not only required impact to rope by RAR’s movement also analysis of the angle and frictional force of ropes and support structures is required. Therefore, rope analysis on structures preceded the development of a control technique for the use of RAR in unstructured buildings.

Therefore, to address the rope-locking problem of the rope, this study analyzed the force generated when the rope comes into contact with the edge of a structure. In general, force analysis research on rope-locking problems has existed before and is still actively ongoing^5–8^. Among them, commonly used methods include analysis rope as group of nodes^5,6^ and finite analysis^7,8^. However, those analysis methods are about force analysis in the rope axial direction regarding the frictional force that occurs when a rope is wrapped around an object different with direction of analysis. In short, there was a requirement of novel analysis that conducted to the direction perpendicular to the direction of rope movement for implement of RIC. Therefore, the rope-locking problem on shear direction according to the robot’s movement was solved through dynamic analysis in this study.

The force analysis of the rope on the edge sets a constraint similar to the situation in which the robot is hung on the rope for application in robot control. When the robot was suspended from the rope, the weight of the robot remained constant, and the tension in the rope did not change. Therefore, the bending angle was used to unlock or move the rope. Force analysis was conducted in a relatively simple two-dimensional and three-dimensional order, and the induction equation of the capstan equation, which is commonly used for rope tension analysis, was used.

The remainder of this paper is organized as follows. In “Mathematical analysis” Section, the analysis is conducted by dividing the definition of the rope contact that occurs when the RAR moves on an unstructured building into two or three dimensions. In “Experimental verification” Section, the planning of the verification experiment and the actual experiment are presented. “Analysis of Experimental results” Section presents an analysis of the experimental results obtained using a fiber rope. Finally, in “Conclusion” Section, the conclusions are drawn.

## Mathematical analysis

### Problem definition

**Figure 2:**
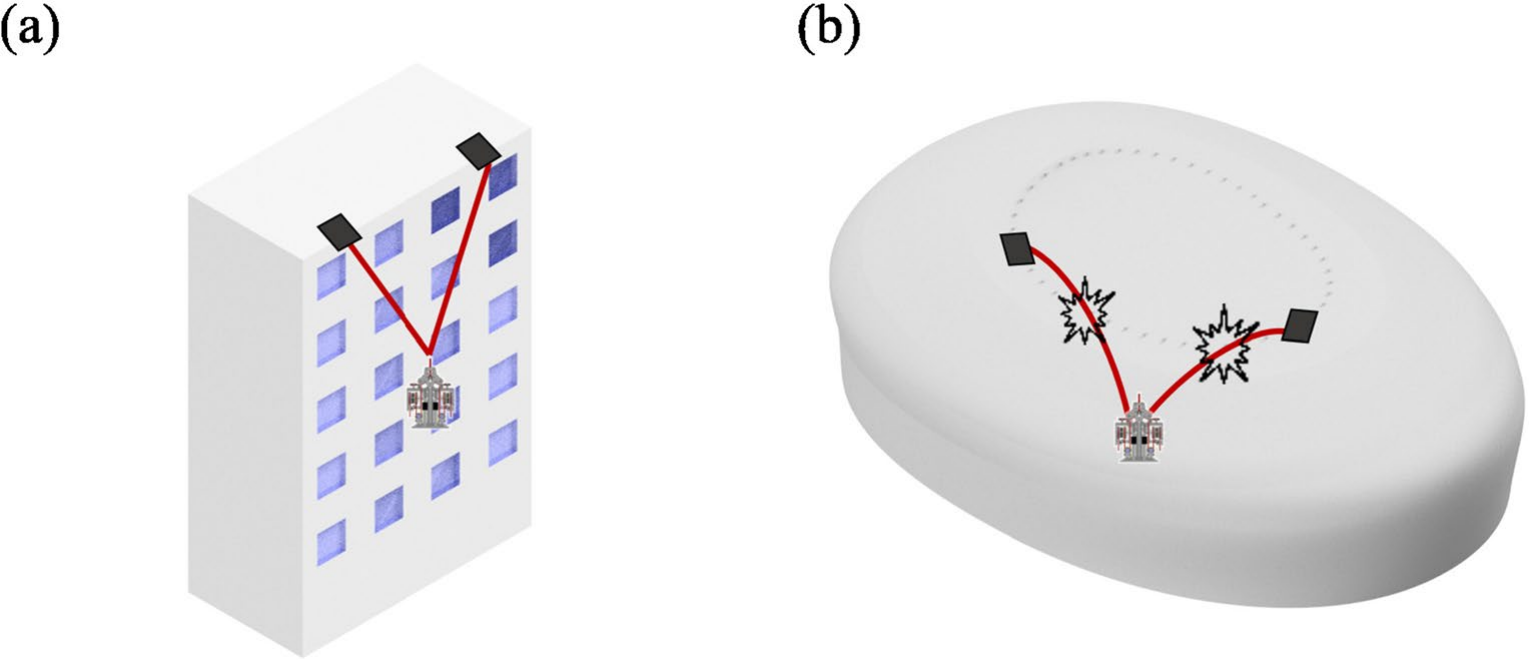
Bent rope scenario of a robot climbing a building. Black line indicates the anchor of rope and the red line denotes the rope. (**a**) Structured building scenario. (**b**) Unstructured building scenario.

The definition of the “rope-locking problem” that occurs when a robot moves on unstructured building should be preceded to analyze the rope characteristics for robot movement. This problem is closely related to the rope characteristics. For the RAR to move on a building, both ends of the rope must be fixed to the building and ascender. When both ends of the rope were fixed, the rope always attempted to be located at the shortest distance between connected endpoints. This rope characteristic is generally not a problem in structured buildings where there are few obstacles between the endpoints when the robot is roped on the building to move to the top of the building. However, in unstructured buildings, a rope is inevitably caught at the edge of an obstacle (Fig. 2). Therefore, in this section, a relatively simple mathematical two-dimensional analysis of the rope-locking problem of the rope was performed. Accordingly, a three-dimensional analysis was then conducted to predict the rope movement in the rope-locking problem for robot control.

### Rope contact for two-dimensional edge

**Figure 3:**
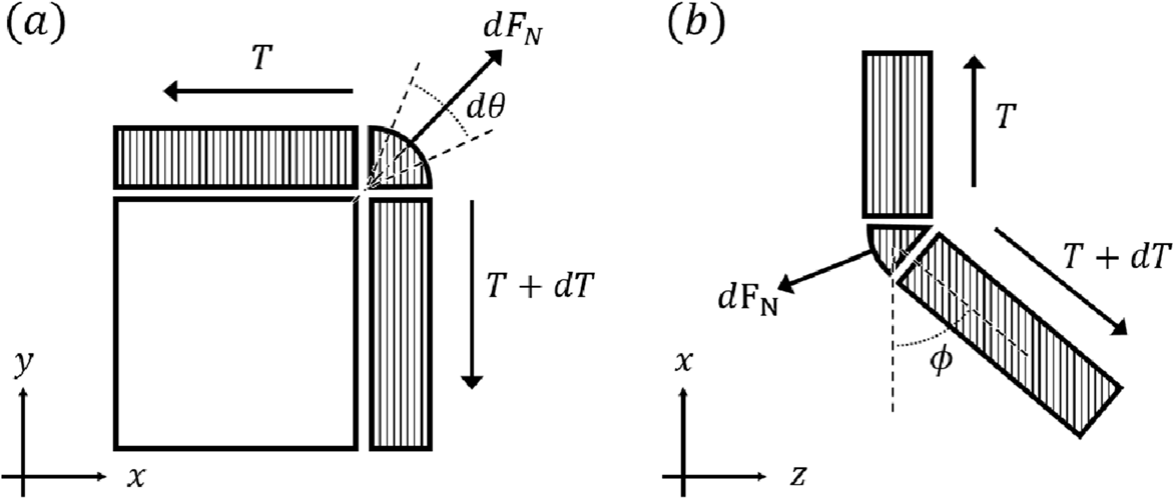
Rope contact situation for a two-dimensional edge. (**a**) Differential analysis at the side view. (**b**) Rope’s moving situation at the edge shown from the top view.

In this section, we analyze the rope-locking problem that occurs when the rope contacts the edge on a plane (Fig. 3a). The rope-locking problem is typically caused by friction on the edge and occurs when the rope is in contact with the edge. Friction is defined as: Friction Coefficient $(\mu )$ *X* Normal force $(F_N)$. If $\mu$ is constant, then friction is affected only by $F_N$^9,10^. $F_N$ is caused by tension at both ends of the rope on rope-locking problem. Both ends of the tension vary according to the capstan equation ($T_{load} = T_{hold} * e^{\mu \theta }$). The rope bending angle $\theta$ in this equation significantly affects the rope-locking problem^11–13^. To analyze the relationship between the bending angle $\theta$ and $F_N$, a differential analysis of the force according to the bending angle of the rope on the x-y surface is conducted considering the situation shown in Fig. 3a. The force equation for the y axis is as follows:

1 $$\begin{aligned} dF_N = (2T+dT)*sin\frac{d\theta }{2} \approx Td\theta \end{aligned}$$

where $d\theta$ is the degree to which the rope is wound around the edge and $dF_N$ is the normal force that the rope receives from the edge.

Further, the rope was hung at the edge at a bending angle of $\theta$, as shown in Fig. 3b. If the rope in the suspended state is pulled at a certain angle, no force is applied by the upper rope tension based on the edge, and only the force due to the lower rope tension exists. Therefore, the force equation for the z axis is as follows:

2 $$\begin{aligned} \mu dF_N = (T+dT)*sin(d\phi ) \end{aligned}$$

where $\mu$ is the friction coefficient between the rope and the edge and $\phi$ is the pulling angle. Finally, the integration of Equations using by Eqs. (1) and (2) is as follows:

3 $$\begin{aligned} \phi (\theta ) = \int _0^{\theta }{\mu }{d\theta } = \mu \theta \end{aligned}$$

Equation (3) implies that even if the rope is caught at the edge, the rope locking at the edge can be moved using only the angle at which the rope is pulled, provided that Please check if this part should be expressed as the friction coefficient $(\mu )$ and the bending angle $(\theta )$.

### Rope contact for n three-dimensional edge

**Figure 4:**
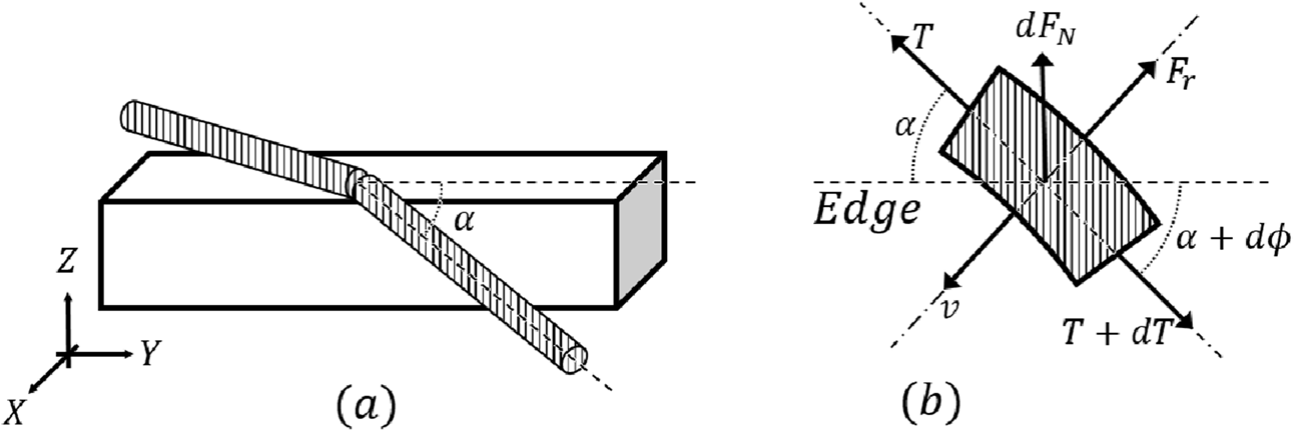
Rope contact situation in the case of three-dimensional edge. (**a**) Rope winding situation from an isometric view. (**b**) Differential analysis from the top view of the rope locked at a three-dimensional edge.

The three-dimensional rope-locking problem is illustrated in Fig.  4a. The appearance of the rope on the edge is different from that in the two-dimensional analysis. Nonetheless, the force is generated in a similar manner. Therefore, the analysis in this section starts similarly to the interpretation in the previous section. However, to simplify the analysis complexity according to the parameters that occur when the rope makes contact, this study assumed two analysis methods for the rope.

- Assumptions
  1. The shape of rope winding is assumed to be helical.
  2. Rope contacts the three-dimensional edge by surrounding it in a circular manner.

Additionally, the analysis of the rope-locking problem involves the rope coming into contact with the edge. Figure 2b depicts the RAR suspended from the end of the rope, and while locking can occur in the robot, this problem is mitigated owing to the constraints of the rope. Because the RAR moves using two ropes, the two ropes are always in a stable state (assumption 1), and the same solution method as the Capstan equation can be applied (assumption 2) by assuming that the rope comes into contact with the edge by encircling it in a circular manner. Thus, the two assumptions were transformed into two conditions.

- Conditions
  1. A certain angle is maintained as long as the rope is hanging from the edge.
  2. Rope bending can be solved similar to the problem of a pulley contacting a three-dimensional edge.

**Figure 5:**
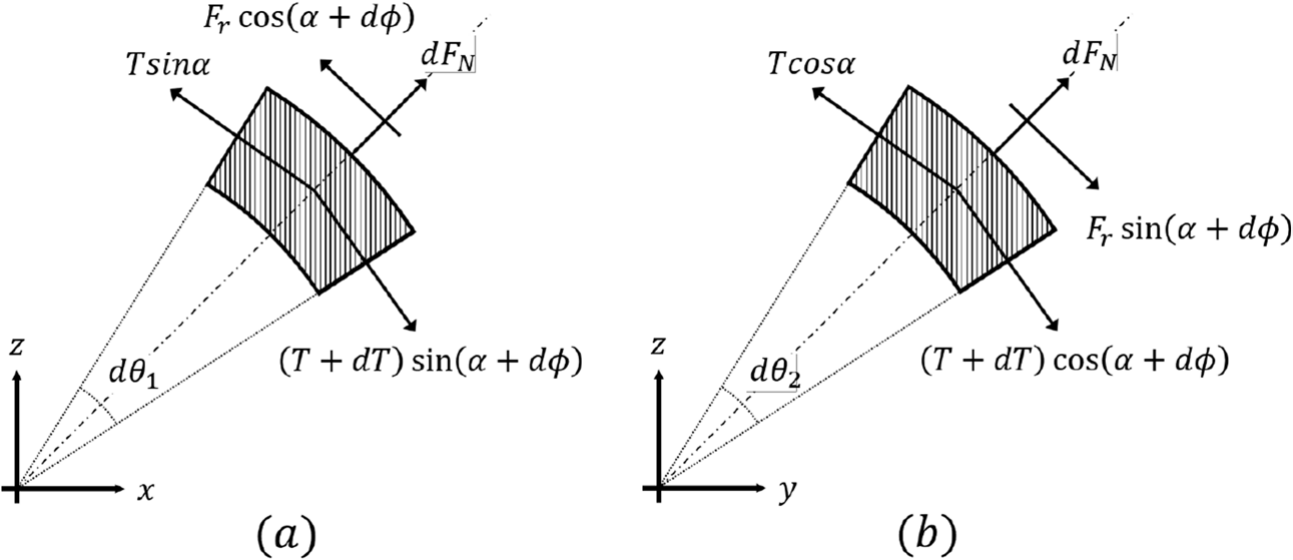
Projection view of rope bending problem. (**a**) On x–z plane, and (**b**) on y–z plane.

The force generated by the rope-locking problem between the rope and the edge in three dimensions is expressed in (Fig. 4b). For ease of expression, global and rope coordinates are expressed as X–Y–Z and x–y–z, respectively. *v* represents the speed of the rope on the edge, and because one end of the rope is fixed as an anchor, the direction is expressed as the y-axis in the rope coordinates (circular motion). $F_r$ denotes the friction between the rope and edge and is expressed as a vector in the opposite direction of motion. $\alpha$ denotes the angle between the edge and rope and $d\phi$ indicates the angle of the rope movement. The problem is difficult to solve because of the variation in the direction of the force. Therefore, to lower the order of the force analysis, the force was analyzed using its projection on the x–z plane and the force equation was solved (Fig. 5a).

#### Sum of forces on X–Z plane

4 $$\begin{aligned} {\left\{ \begin{array}{ll} X: dTsin\alpha + Td\phi *cos\alpha - F_rcos(\alpha + d\phi ) = 0 \\ Z: dF_N \approx Td{\theta }_1*sin\alpha \end{array}\right. } \end{aligned}$$

where ${d\theta }_1$ denotes the rope-bending angle in the X–Z plane.

However, there are several variables that can be used to solve the force equation using only Eq. (4). Therefore, the force equation can be solved by simplifying it, which can be achieved by projecting the force onto the Y–Z plane to reduce the number of variables, as illustrated in Fig. 5b.

#### Sum of forces on Y–Z plane

5 $$\begin{aligned} {\left\{ \begin{array}{ll} Y: dTcos\alpha - Td\phi *sin\alpha + F_rsin(\alpha + d\phi ) = 0 \\ Z: dF_N \approx Td{\theta }_2*cos\alpha \end{array}\right. } \end{aligned}$$

where ${d\theta }_2$ denotes the rope-bending angle in the Y–Z plane. By combining X-axis equation in Eq. 4 X and Y-axis equation in Eq. 5, we obtain Eq. (6) as follows:

6 $$\begin{aligned} F_r = Td\phi \end{aligned}$$

$F_r$ follows the Coulomb friction. Furthermore, substituting Z-axis equation in Eq. (4), the final equation can be obtained as

7 $$\begin{aligned} d\phi = {\mu }*sin\alpha *{d{\theta }_1} \end{aligned}$$

where $\mu$ denotes the total friction coefficient between the edge and rope. Finally, integration must be performed, and an independent judgment between the variables is inevitably required to solve 7. However, $\phi$, $\alpha$ and ${\theta }_1$ are independent of the changes in each variable integrated over the variables individually, we obtain:

8 $$\begin{aligned} \phi (\theta , \alpha ) = \int _0^{\theta }{\mu }*sin\alpha *{d{\theta }_1} = {\mu }{\theta }sin\alpha \end{aligned}$$

Equation (8) shows that regardless of the amount of tension applied to the rope, rope locking can occur on a three-dimensional edge. This result is similar to that expressed in Eq. (3) in the two-dimensional analysis. However, the rope is influenced by gravity and exists only below the edge. Therefore, the rope-locking problem can be solved under the constraint $\alpha + \phi \le 180^{\circ }$.

## Experimental verification

**Figure 6:**
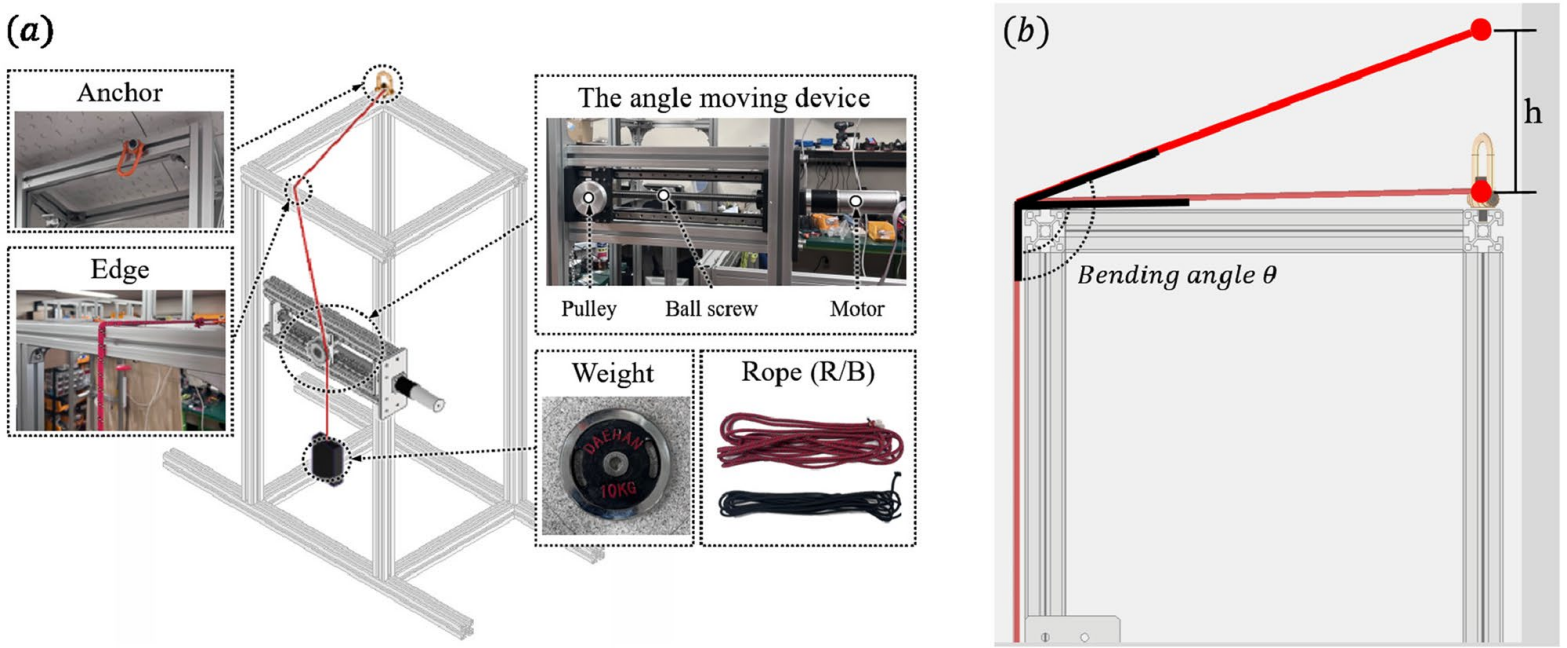
3D graphic image of testbench. (**a**) Anchor position is variable. Edge material is basically aluminum and can be switched to change the friction coefficient. The intended role of the pulley is to move rope position on edge without changing the tension. The lead of the ball screw is 3mm/rev. Two types of ropes were used in this experiment. (**b**) Bending angle $\theta$ was changed by changing h ($\theta = 90^{\circ }$, h = 0mm & $\theta = 127^{\circ }$, h = 430mm).

Experimental verification was conducted to determine if the force analysis of rope-locking problem performed in Section 2 on two- and three-dimensional edges is accurate. To create a situation similar to that in which the equations were solved, a verification experiment was performed for each equation. A testbench was used to perform verification experiments. The testbench was largely composed of an anchor and an angle-moving device (Fig. 6a). The anchor was installed at the end of the testbench such that the rope gets locked during its installation. The angle-moving device consisted of a rope pulley, a motor, and a ball screw, which allowed the rope pulley to move in both directions(Fig. 6a).

In addition, a unique method was used to set variables used in the equations as this could not be achieved solely using the testbench. Experiments were conducted by changing the rope type to vary the friction coefficient $\mu$. Two types of ropes were used in the experiment: red rope ($\mu = 0.081$) and black rope ($\mu = 0.11$) (Fig. 6a). Except for different surface roughness values, the two ropes had identical structures with a radius of $6\pi$. To set the bending angle $\theta$, experiments were conducted by varying the height of the anchor to determine $\theta$. For this experiment, the values of $\theta$ used were $90^{\circ }$ and $127^{\circ }$ that selected as smallest and largest, which assumed the contact scenario between the rope and edge. This was accomplished by adjusting the height of the anchor, as shown in Fig. 6b.

**Figure 7:**
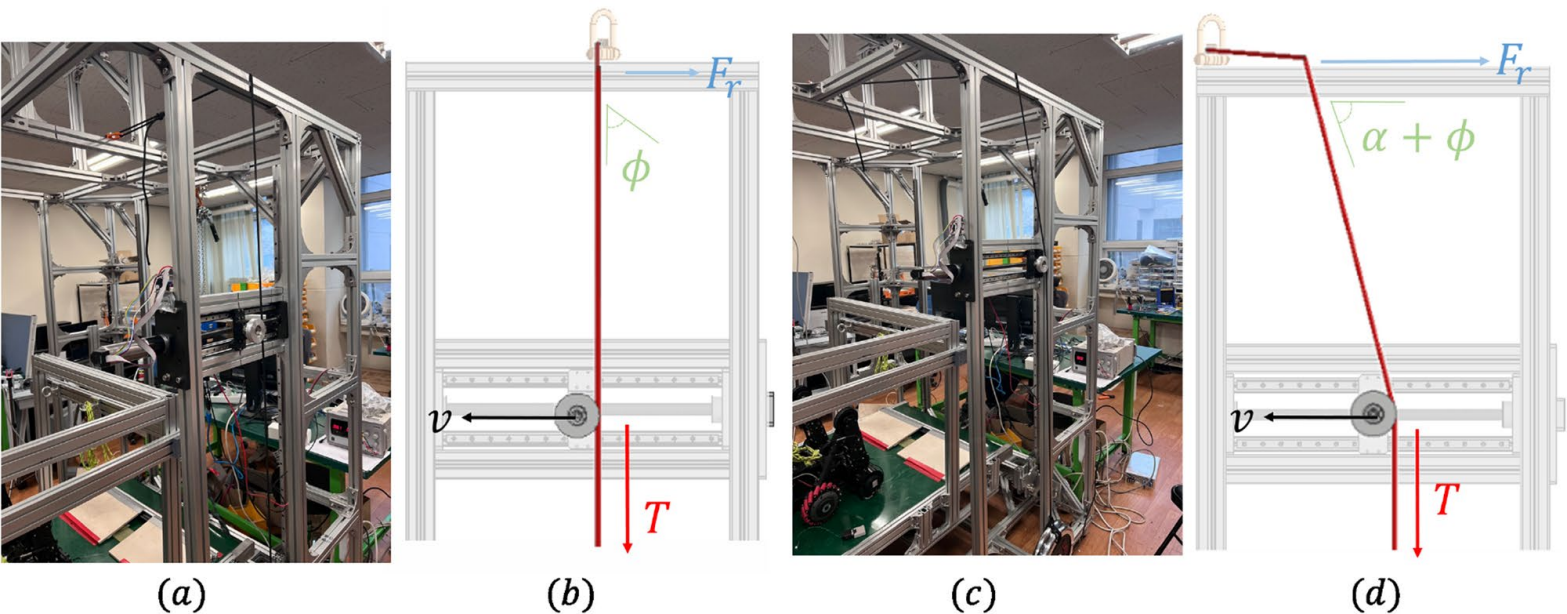
Experiment of rope-locking problem. (**a**) Experimental setup of the two-dimensional edge scenario. (**b**) Illustration of the experiment using a 3D graphic: T denotes rope tension due to weight $(= mg*sin\phi )$, $F_r$ denotes rope friction at its point of contact with the edge, and *v* denotes rope’s moving velocity. (**c**) Experiment setup in the three-dimensional edge scenario.(**d**) Illustration of the experiment using 3D graphic: T denotes rope tension due to weight $(= mg*sin(\alpha +\phi ))$, $F_r$ denotes rope friction at its point of contact with the edge, and *v* denotes the moving velocity of the rope.

Before proceeding with the experiment, an initial setup was required. In both experiments, the rope was hung in the order of an anchor-edge rope pulley to create a bending angle, and a weight was attached to the end of the rope. The weight was set to 20 kg (10 kg $\times$ 2), which was the weight of the RAR. However, the two-dimension and three-dimension experiments require different angles when a rope is caught on an edge. In the two-dimensional edge scenario, the initial setting of the angle of anchor edge weight should be $\phi = 0^{\circ }$ (Fig. 7a,b), whereas for the three-dimensional edge scenario, it should be $\phi = \alpha$ (Fig. 7c,d). After this initial setup, in each experiment, the ball screw was moved using a motor in the direction in which the angle of the anchor-edge weight increased, and $\phi$ was measured precisely at the moment when the rope was unlocked at the edge. Each same experiment was repeated five times to reduce uncertainty.

## Experimental result

**Figure 8:**
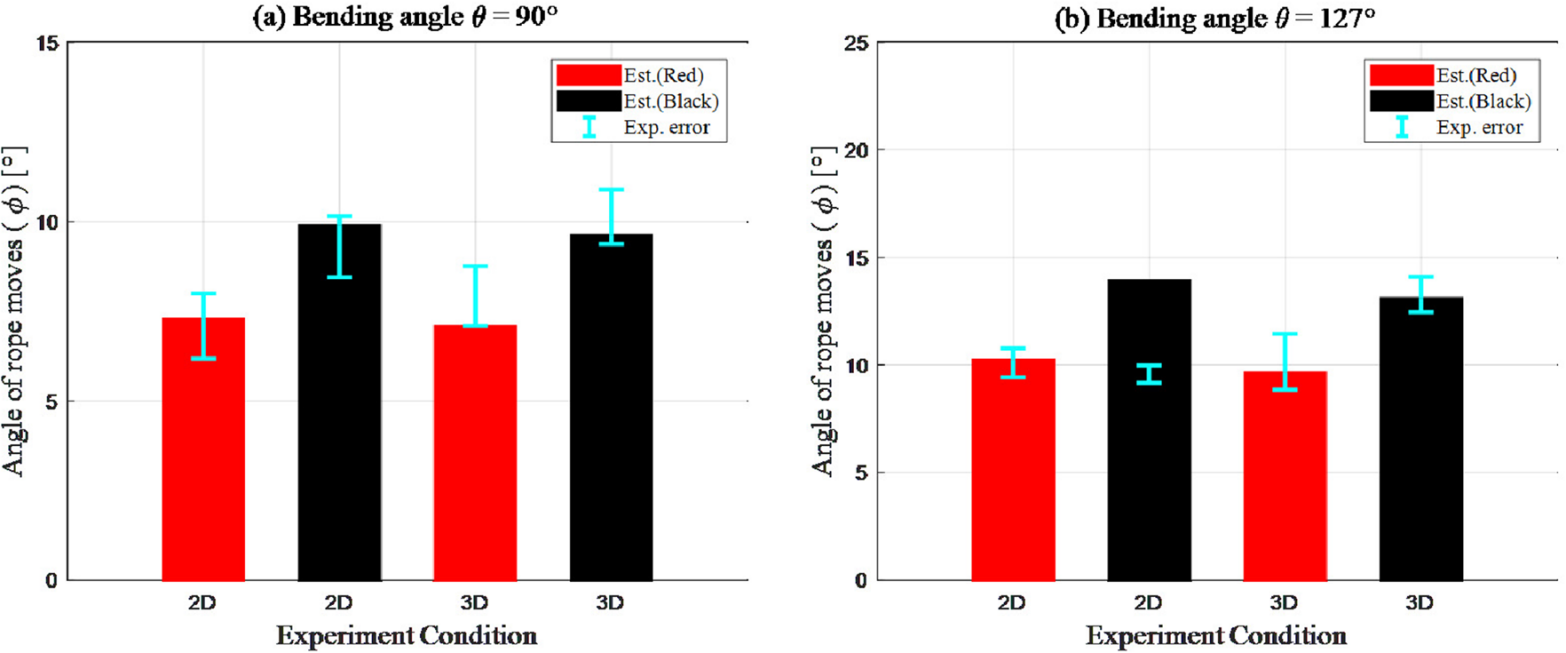
Error graph of estimated value compare with experiment value. Graph colors denote the rope type used in experiment.

The resulting graph was divided into two graphs according to the bending angle $\theta$. The estimated value was denoted using a bar graph, and the error between the estimated and experimental values is denoted by an error graph above the bar (Fig. 8 and Table 1). Because it is difficult to measure the friction coefficient $\mu$ according to the rope type, and to compare the estimated and experimental values to verify the force analysis, we assumed friction coefficient values of 0.081 and 0.11 for the red and black ropes, respectively^9,10^.

### Case 1: Bending angle $\theta = 90^{\circ }$

First, the experimental verification results of the proposed force analysis method in the case of “locking problems” with a bending angle of $\theta = 90^{\circ }$ show similar results for the estimated values (Eqs. 3 and 8) and experimental values (Fig. 8a). Examining the results from the error perspective of the estimated and experimental values, the “locking problem” on the bending angle $\theta = 90^{\circ }$ tends to result in higher error values in two-dimensional edge scenarios compared to those in three-dimensional edge scenarios ($0.15^{\circ }$–$0.19^{\circ }$). This error is caused by the difference in the constraint conditions, and it is obvious that the three-dimensional edge scenario is more vulnerable to the collapse of the force equilibrium than its two-dimensional counterpart.

Additionally, analyzing the results according to the rope type, a lower error was observed in the case of black rope compared to that obtained for the red rope, which showed a maximum error of $0.15^{\circ }$ between the results in the three-dimensional edge scenario. However, the tendency of the result according to the friction coefficient $\mu$ (rope type) is similar, and the error between the estimated and experimental values is in acceptable range. Therefore, it can be considered as an uncertainty caused by the rope characteristics.

### Case 2: Bending angle $\theta = 127^{\circ }$

Further, the experimental verification results obtained in the case of ”locking problems” with a bending angle of $\theta = 127^{\circ }$ are shown in Fig. 8b. The overall results were similar to that observed in the case of $\theta = 90^{\circ }$. Under all conditions, the estimated and experimental values were similar, and fewer errors occurred in the case of black rope compared to those observed when using the red rope. However, a singularity of the result occurred in the two-dimensional edge case using a black rope. The average of the experimental values was only $9.65^{\circ }$, whereas the estimated value was $13.92^{\circ }$. This result indicates that Eq. 3 was incorrect, owing to which the error rate exceeded $30.6\%$.

**Table 1:** Comparison between estimated & experimental values.

Locking scenario	Bending angle $\theta = 90^{\circ }$		Bending angle $\theta = 127^{\circ }$	
	$\mu = 0.081$ (Red)	$\mu = 0.11$ (Black)	$\mu = 0.081$ (Red)	$\mu = 0.11$ (Black)
Two-dimensional	$7.29^{\circ } (\pm 13\%)$	$9.9^{\circ } (\pm 9\%)$	$10.25^{\circ } (\pm 7\%)$	$13.92^{\circ } (N/A)$
Three-dimensional	$7.09^{\circ } (\pm 12\%)$	$9.62^{\circ } (\pm 8\%)$	$9.64^{\circ } (\pm 14\%)$	$13.10^{\circ } (\pm 7\%)$

### Singularity analysis

Various factors are responsible for the error rates of the estimated and experimental values for bending angle $\theta = 127^{\circ }$, in the case of two-dimensional edge using black rope. However, considering that the range of the error between the estimated and experimental values was $9.25^{\circ }$ to $10.06^{\circ }$ and that the equation yielded accurate results when the bending angle $\theta =90^{\circ }$, it can be concluded that the issue lies with the friction coefficient $\mu$. Therefore, further experiments were conducted by fixing the experimental conditions in two dimensions: the black rope condition and changing the bending angle $\theta$ between $90^{\circ }  \&  127^{\circ }$.

**Figure 9:**
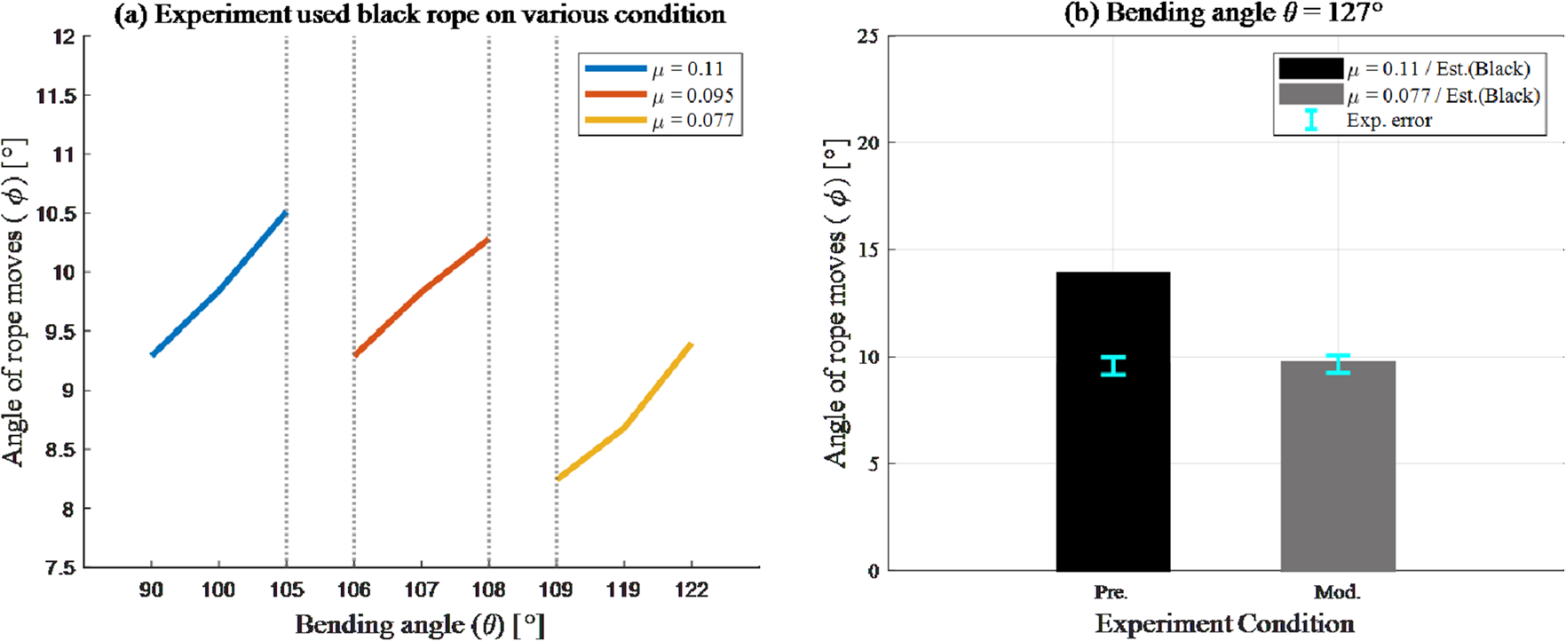
Singularity analysis. (**a**) Results of additional experiments. Inclinations of three-graphs were approximately 0.61, 0.5, and 0.58, which implies that they have same tendency on “locking problem.” (**b**) Modified estimated value and comparison with experiment value.

The results of these experiments are shown in Fig. 9a. The graphs obtained have different shapes despite using the same rope for the experimental values. The slopes of the three graphs are similar and it was confirmed that the results had a tendency to vary depending on the bending angle $\theta$. The tendency checks confirmed that it behaved differently depending on the bending angle $\theta$. Therefore, the friction coefficient $\mu$ at the bending angle $\theta = 127^{\circ }$ was modified to 0.077, which is equivalent to the experimental value provided in Fig. 9b and Table 2. After modification, the estimated value was $9.74^{\circ }$, and the error from the experimental value was in the range of $\pm 4\%$.

**Table 2:** Comparison between modified estimated value the resultant & experimental value.

Locking scenario	Bending angle $\theta = 127^{\circ }$	
	$\mu = 0.11$ (Pre.)	$\mu = 0.077$ (Mod.)
Two-dimensional	$13.92^{\circ } (N/A)$	$9.65^{\circ } (\pm 4\%)$

### Analysis of experimental results

**Figure 10:**
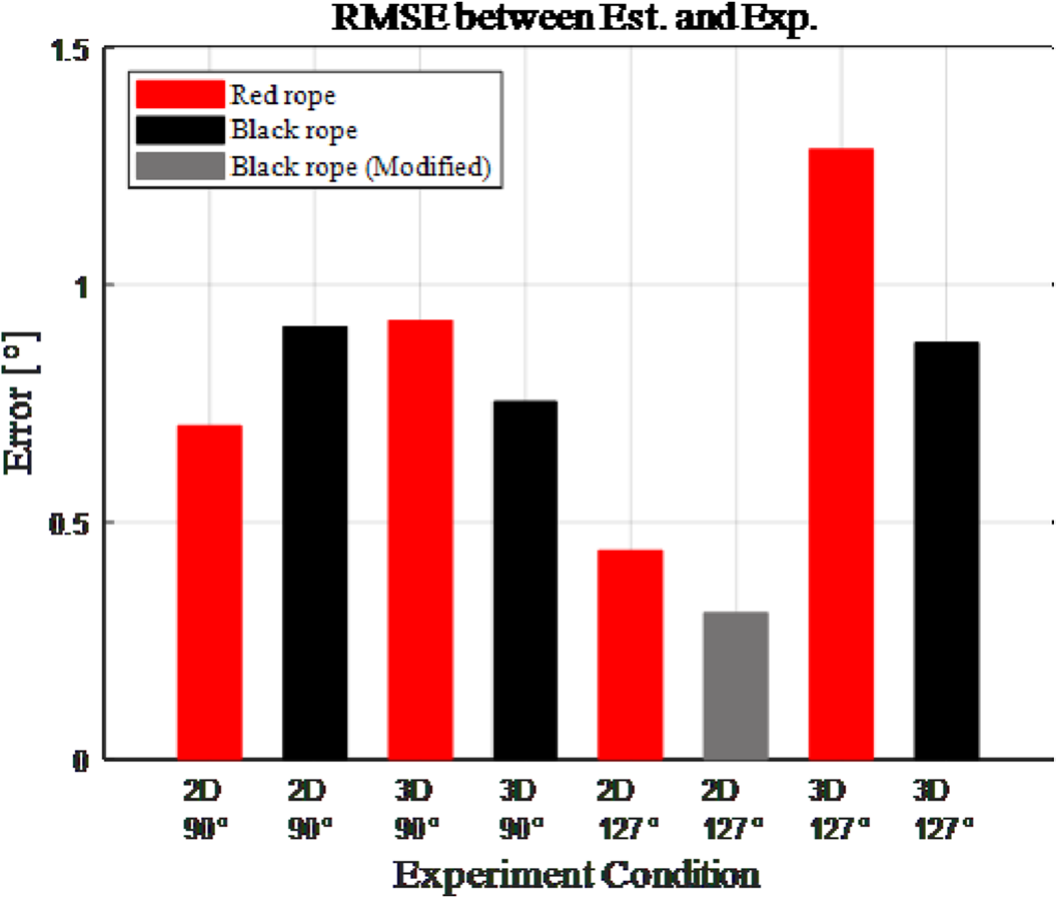
RMSE of experiment.

To more accurately examine the difference between the estimated and experimental values, the values were compared using the root-mean-square error (RMSE). The RMSE of the angle of rope movement $\phi$ was calculated based on Eq. (9) and is plotted in Fig. 10. Overall, a slight difference was observed between the experimental and estimated values obtained by force analysis. However, the synthetic rope used in the experiment was a time-varying system, that is, its characteristics often changed over time^14–16^. Considering these properties, an error of less than $1.5^{\circ }$ between the estimated and experiment values increases the reliability of the estimation accuracy. Therefore, the force analysis relationship between rope and edge (Eqs. 3 and 8) was confirmed to be accurate, and the rope-locking problem can be solved if angle of rope $\phi$ can be changed whatever their condition is.

9 $$\begin{aligned} RMSE = \sqrt{\sum _{i=1}^5 \frac{1}{5}*({\phi }_{estimation}-{\phi }_{Real})^2} \end{aligned}$$

## Conclusion

This study presents a method for creating an external wall working robot that utilizes rope by conducting a force analysis on the rope-locking problem that may arise during its operation. The term rope-locking problem refers to a situation where the tensioned rope becomes trapped by a structure. Through force analysis, it was determined that the rope could be moved at an angle $\phi$, regardless of the applied tension. The calculated equation was verified by experiment on the testbench, which was created assuming that the rope is hung on the robot. When comparing the estimated and experimental values with RMSE after the verification experiment, it was confirmed that errors of less than $15\%$ (red rope) and less than $10\%$ (black rope) occurred according to the rope type. However, because the RMSE size is not large ($<1.3^{\circ }$), it was judged to be uncertain due to the characteristics of the synthetic fiber rope used, and the rope-locking problem was concluded to be solved.

Therefore, as a follow-up to this study, a motion control algorithm will be developed and implemented to address the rope-locking problem, and to ensure the smooth operation of the RAR exterior wall working robot. By applying the corresponding motion control algorithm, obstacles in the application of an exterior wall-working robot, such as RAR, which utilizes ropes for climbing unstructured buildings, can be eliminated. Moreover, if the constraints utilized in this study are generally applied to analyze the forces required to resolve the rope-locking problem, its application may not be limited to robots that utilize ropes, and can assist in addressing movement disturbance in studies related to cranes and winches.

## Data Availability

All data generated or analysed during this study are included in this published article.
